# A Food Handler-Associated, Foodborne Norovirus GII.4 Sydney 2012-Outbreak Following a Wedding Dinner, Austria, October 2012

**DOI:** 10.1007/s12560-013-9127-z

**Published:** 2013-09-12

**Authors:** Sabine Maritschnik, Elisabeth Eva Kanitz, Erica Simons, Marina Höhne, Heidelinde Neumann, Franz Allerberger, Daniela Schmid, Ingeborg Lederer

**Affiliations:** 1 Austrian Agency for Health and Food Safety (AGES), Währingerstraße 25a, 1096 Vienna, Austria; 2 European Programme for Intervention Epidemiology Training (EPIET), European Centre for Disease Prevention and Control (ECDC), Tomtebodavägen 11A, 17165 Solna, Sweden; 3 FG15 Molecular epidemiology of viral pathogens, National Consultant Laboratory for Norovirus, Robert Koch Institute, Nordufer 20, 13353 Berlin, Germany; 4 Public Health Authority Salzburg, Sebastian-Stief-Gasse 2, 5010 Salzburg, Austria

**Keywords:** Novel norovirus GII.4 variant, Hygiene violations, Gender-specific risk, Outbreak investigation

## Abstract

On October 12, 2012, the provincial public health directorate of Salzburg reported a suspected norovirus (NV) outbreak among guests of a wedding–reception. The investigation aimed to confirm the causative agent, to identify the mode of transmission and to implement appropriate preventive measures. A probable outbreak case was defined as a wedding guest with diarrhoea or vomiting with disease onset from 7 to 10 October 2012 and who consumed food at the wedding dinner prepared by a hotel in the province Salzburg on 6 October 2012. A confirmed outbreak case fulfilled the criteria of a probable outbreak case and had a laboratory-confirmed NV infection. We conducted a cohort-investigation among the wedding guests. The case definitions were fulfilled in 26 wedding guests (25 %) including 2 confirmed cases. Females were 3.2 times more likely to develop disease (95 % CI 1.4–7.2) as compared to males. A mushroom dish was found to be associated with disease risk among females (risk ratio 2.3, 95 % CI 1.2–4.3). Two of 2 tested case-patients and 6 of 14 kitchen workers tested were positive for NV GII.4 Sydney. One kitchen staff-member worked during the wedding dinner despite diarrhoea. No food safety training was documented for the employees and the kitchen staff’s restroom was lacking operational facilities for hand hygiene. We report the first investigated outbreak due to GII.4 Sydney, which was likely due to a symptomatic kitchen worker. Gender-specific eating behaviour may have posed female guests at higher risk of NV infection.

## Introduction

Norovirus (NV), a calicivirius, is now recognized as the leading cause of gastroenteritis and of food-related outbreaks in the industrialized world (CDC 2011; Van Beek et al. 2013; Maunula et al. 2009). Currently, 5 genogroups (GI–GV) and at least 34 genotypes are known. NV infection in humans is caused primarily by GI and GII, and during the past two decades most outbreaks in the industrialized world were due to variants of genotype GII.4. New GII.4 variants have emerged every 2–3 years, replacing previously predominant GII.4 strains and often resulting in increased outbreak activity (Siebenga et al. 2007).

In late 2012, Australia, New Zealand and Japan, followed by some European countries (France, the Netherlands, Scotland, United Kingdom), reported the emergence of a new variant of genotype GII.4, the NV GII.4 Sydney 2012 (Van Beek et al. 2013; Bennett et al. 2013; Fonager et al. 2013; CDC 2013).

On 12 October 2012, the provincial public health directorate informed the Austrian Agency for Health and Food Safety (AGES) about a cluster of at least ten cases of gastroenteritis among attendees of a wedding dinner at hotel A in the Austrian province Salzburg. The stool specimen from one attendee, who fell ill with diarrhoea within 2 days after the wedding, tested positive for NV by reverse-transcriptase (RT)-PCR. The provincial public health directorate of Salzburg mandated AGES to confirm the suspected NV outbreak and to identify the source of infection in order to set appropriate measures for preventing future NV-outbreaks in such settings.

## Methods

### Descriptive Epidemiology

We defined a probable outbreak case as (1) a wedding guest with diarrhoea or vomiting with disease onset from 7 to 10 October 2012 and (2) who consumed food at the wedding dinner prepared by hotel A in the province Salzburg on 6 October 2012. A confirmed outbreak case fulfilled criteria (1) and (2) of a probable outbreak case and had a laboratory-confirmed NV infection. Active case finding was conducted by the district public health office by telephone and personal interviews with the wedding guests, whose contact details were provided by the bridegroom’s father. Data on age, sex and date of disease onset were collected from all identified cases. We performed hypothesis-generating trawling interviews with the first ten cases registered at the district public health office.

### Analytical Epidemiology

Findings of the descriptive epidemiological investigation led us to suspect food items offered at the wedding dinner as the source(s) of infection. To test this hypothesis, we conducted a retrospective cohort investigation. All guests of the wedding dinner at hotel A on 6 October 2012, who had possibly consumed food prepared in the hotel kitchen, were eligible for inclusion to the cohort investigation. The cohort participants received a standardized questionnaire by mail from AGES. We contacted non-responders after two weeks by telephone and performed a personal interview. We collected information from all cohort participants on age, sex, disease status and disease onset defined as acute gastroenteritis (i.e. diarrhoea and/or vomiting) occurring within 48 h following the wedding dinner. For each cohort participant we ascertained information on the consumed dishes at the wedding dinner, and compared food-specific attack-rates among exposed and unexposed and calculated food-specific risk ratios with 95 % confidence intervals (CI). Data were entered into EpiInfo Version 7. All statistical analyses were performed by using STATA 11 (StataCorp).

### Laboratory Investigations

Stool specimens from two wedding guests, who fell sick with gastroenteritis within 48 h following the wedding dinner and from all 14 kitchen workers and 2 hotel employees were tested for bacterial enteric pathogens as described elsewhere (Huhulescu et al. 2009) and for NV by using realtime RT-PCR (Bernard et al. 2013). Genotyping was performed on NV RNA from the two positive wedding guests and the six kitchen workers by the German Consultant Laboratory for NV. Processing, amplification and sequencing of region A [(RNA-dependent RNA polymerase gene (ORF1))] and region C in the 5′-terminal part of the capsid gene (ORF2) were performed as described elsewhere (Felsenstein 1989). Sequences were aligned to prototype sequences drawn from GenBank with use of CLUSTAL W, version 1.6, and phylogenetic trees were produced using the neighbour joining and DNADIST program of the Phylogeny Interference Package (PHYLIP), version 3.57c (Felsenstein 1989). In addition, NV strains from previous Austrian outbreaks, which indicated the genotype NV GII.4 Sydney 2012 by routine genotyping performed by the Austrian Reference Laboratory, as described elsewhere (Oh et al. 2003), were selected for re-genotyping by the German Consultant Laboratory for NV.

### Environmental Investigations

Food inspectors performed an inspection of the hotel kitchen on 11 October 2012. At this time, no food leftovers from the wedding dinner were available for microbiological testing. Since ingredients of the wedding dessert included berries, the food inspectors collected samples from commercially produced, frozen mixed berries and a berry cocktail in two originally sealed 2.5 kg bags and from a self-produced, frozen berry ragout, all stored at the hotel kitchen refrigerator. We ascertained the work schedule of the kitchen staff for the week in which the wedding dinner took place and asked whether they were sick with gastroenteritis within 14 days before or during the outbreak. We also interviewed the hotel manager about the tasks of every kitchen employee.

## Results

### Descriptive Epidemiology

**Fig. 1:**
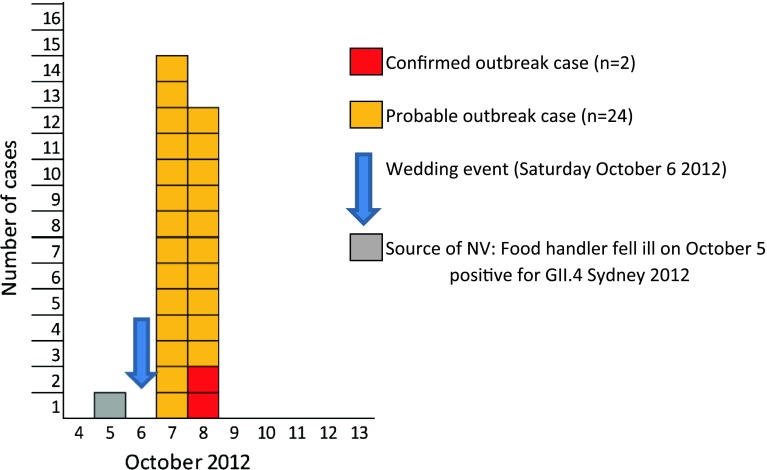
NV outbreak cases by date of onset following a wedding dinner, Salzburg, October 2012 (*N* = 26)

We identified 26 outbreak cases among a total of 103 wedding guests (overall attack rate 25 %), including 2 confirmed outbreak cases. The median age of the wedding guests was 30.5 years (range 5–86 years) and the female to male ratio 1:1. Among the outbreak cases, the median age was 37 years (range 7–81 years) and the female to male ratio 3.3:1 (20 female cases). None of the 26 cases required hospitalization or had fatal outcome. The outbreak took place on 7 and 8 October 2012, with the majority of cases with onset of disease October 7 (Fig. 1). The pattern of the outbreak curve indicated a point source. All outbreak cases were residents of the Austrian province Salzburg. The trawling interviews with the ten cases revealed exposure to food served at the wedding dinner as the only common link and excluded a vomiting incident at the wedding dinner as another possible point source.

### Analytical Epidemiology

Out of the 103 guests who attended the wedding dinner, 90 guests agreed to participate in the cohort investigation (response rate 87 %). The median age of the participants was 30.5 years and 46 (51 %) were female. Females were 3.2 times more likely to develop the disease (95 % CI 1.41–7.19; *p* value = 0.002), as compared to males. Compared with unexposed, guests who ate the wedding cake were 2.2 times more likely to develop disease (95 % CI 0.9–5.3; *p* value = 0.053). The wedding cake was eaten by 21 of the 26 (80.8 %) cases (Table 1). Participants who consumed the mushroom dish had a 2.3 times higher risk of developing the disease (95 % CI 1.25–4.35; *p* value = 0.008), as compared with those who did not eat the mushrooms. When stratified by gender, the relative risk of developing disease following mushroom consumption was 2.3 (95 % CI 1.21–4.34; *p* value = 0.012) among female participants versus 1.5 (95 % CI 0.32–7.10; *p* value = 0.612) among male cohort participants, but which was no longer statistically significant. The mushroom consumption-attributable fraction among the exposed female participants was 56 % (95 % CI 17–77). Among all female participants, 31 % of the incidence NV gastroenteritis following the wedding dinner could be attributed to the consumption of the mushroom dish. The joint effect of being female and exposed to the mushroom dish (*R*_observed_ = 0.68) was greater than the combined independent effects of being female and having consumed the specific dish (*R*_expected_ = 0.33), which indicate a biological interaction between the two exposure factors. A poisson regression model including the interaction term for gender and consumption of mushrooms revealed that females exposed to the mushroom dish were 5.7 times more likely to develop the disease (95 % CI 1.81–17.81; *p* value = 0.003) as compared to unexposed males. The relative risk of disease associated with wedding cake consumption among female and male participants did not remain statistically significant after stratification (RRfemale: 2.5; 95 % CI 0.86–7.12, *p* value = 0.046; RRmale: 1.3; 95 % CI 0.26–6.15; *p* value = 0.774).

**Table 1:** Gender- and food-specific attack rates (AR %) and risk ratios following a wedding dinner, Salzburg, October 2012

Exposure	Exposed			Unexposed			Risk ratio	95 % CI	*p*
	Total	Cases	AR (%)	Total	Cases	AR (%)			
Gender (female)	46	20	43.48	44	6	13.64	3.19	1.41–7.19	0.002
Menu 1	63	18	28.57	27	8	29.63	0.96	0.48–1.94	0.919
Vegetables	61	17	27.87	29	9	31.03	0.9	0.46–1.77	0.757
Pork medallion	65	19	29.23	25	7	28	1.04	0.50–2.17	0.908
Menu 2	24	9	37.5	66	17	25.76	1.46	0.75–2.81	0.277
Vegetables	24	9	37.5	66	17	25.76	1.46	0.75–2.81	0.277
Tagliatelle	24	9	37.5	66	17	25.76	1.46	0.75–2.81	0.277
Fish filet	25	9	36	65	17	26.15	1.38	0.71–2.67	0.356
Mushrooms	27	13	48.15	63	13	20.63	2.33	1.25–4.35	0.008
Hot raspberries	77	21	27.27	13	5	38.46	0.71	0.33–1.54	0.410
Chocolate souffle	80	22	27.5	10	4	40	0.69	0.30–1.59	0.411
Goulash soup	32	10	31.25	58	16	27.59	1.13	0.58–2.20	0.714
Wedding cake	59	21	35.59	31	5	16.13	2.21	0.92–5.28	0.053

### Laboratory Investigations

NV was detected in stool specimens collected from the 2 tested outbreak cases and in stool specimens from 6 of the 14 kitchen workers. For all NV confirmed cases, the NV GII.4 variant Sydney 2012 was identified.

The re-genotyping of NV outbreak strains revealed that NV GII.4 Sydney was implicated in a small hospital-outbreak in February 2012 and a family-outbreak in November 2012. Overall, NV GII.4 Sydney accounted for 18 % of all NV strains sequenced in 2012 and was not detected in Austria before 2012.

### Environmental Investigations

All three samples from frozen mixed berries, berry cocktail and mixed berry ragout tested negative for NV. According to the hotel manager, the specific mushroom dish was prepared on the morning of the wedding dinner and subsequently reheated just before serving. Parsley was added by hand after heating and prior to serving. One of the six NV positive kitchen workers had already fallen ill with diarrhoea on October 5 and continued to work in the hotel kitchen on October 5 and 6 (the day of the wedding). His main tasks included cleaning flatware and dinnerware that was used for the wedding dinner. According to the official report of the food inspector, the kitchen staff’s restroom was lacking operational facilities for appropriate hand hygiene.

## Discussion

On 7 and 8 October 2012, a NV outbreak occurred including 26 cases among guests of a wedding dinner on October 6. Female participants were three times more likely to develop the gastroenteritis than males. Our findings were in line with findings of an analysis of 60 NV outbreaks of gastroenteritis in Catalonia in 2004–2005. Women were at a 1.4 times higher risk of NV infection than men (Arias et al. 2010).

In the early 2000s, a predominance of females among outbreak cases of viral gastroenteritis was already described in Australia and the U.S. (Marshall et al. 2005; Fankhauser et al. 2002). In a study on gender-differences in eating behaviour among Austrian adolescents, Askovic et al. (2012) found that boys preferred meat and fast food while girls consumed fruits, vegetables and healthy food more frequently. A German population-based study also showed that men are more likely to eat energy dense foods, red meat, sausages, whereas women are more likely to consume fruits, vegetables, foods high in carbohydrates and dairy products (Fekete et al. 2012). This gender-specific eating behaviour may pose women at a higher risk of consuming biological plausible dishes that are contaminated with enteric pathogens, compared with men. Dishes commonly involved in NV foodborne outbreaks are lettuce, fresh fruits and uncooked vegetarian food or dishes manually manipulated after cooking (Li et al. 2012). The only wedding dish found to be associated with the risk of NV gastroenteritis was a mushroom dish, decorated with parsley that was added after heating. In our outbreak, 35 % (16/46) of the female cohort participants compared to only 25 % (11/44) of the male participants consumed this mushroom dish. Surprisingly, after stratification by gender the consumption of mushroom dish remained significantly associated with the disease risk only among female participants. The joint effect of being female and exposed to the mushroom dish was greater than the combined independent effects of being female and having consumed the specific dish, which indicates a biological interaction (i.e. synergy) between these two exposure factors. A possible explanation for this finding may be that the female preference of ‘lighter dishes’ prompted them to take more portions from the mushroom dish, which could have increased their risk of getting a mushroom portion contaminated with NV, as compared to males. Including questions on the number of portions females and males had eaten, would have made it possible to test this hypothesis.

The outbreak agent was identified as the newly emerging NV GII.4 Sydney strain (Van Beek et al. 2013). This new NV GII.4 variant was first documented in Australia in March 2012. In the United Kingdom, an early onset of the 2012 winter NV season was ascribed to the emergence of this strain (HPA 2012). In the United States, GII.4 Sydney has spread rapidly nationwide, causing an increasing number of outbreaks in 2012 (CDC 2013). In Austria, in 2010, 2011, and 2012 NV GII.4 2010 was predominant, and NV GII.4 Sydney was not detected before 2012 (Springer 2009; Lederer and Springer 2010, 2011, 2012). In contrast to the observations made in Scotland, Denmark and in the U.S. (Bennett et al. 2013; Fonager et al. 2013; CDC 2013), we did not observe an overall increase in the NV activity in 2012 due to the emergence of NV GII.4 Sydney.

Based on a systematic literature review, Desai et al. (2012) found that outbreaks due to NV GII.4 strains were associated with more severe outcomes defined as higher rates of hospitalization and case-fatality. Observations from the NV GII.4 Sydney outbreaks in the United Kingdom and United States do not suggest increased virulence of this new variant (HPA 2012). These are in line with our findings from the wedding outbreak investigation, in which none of the 26 cases required hospitalization or had fatal outcome. In contrast, Japan reported higher rates of hospitalization in elderly patients with gastroenteritis due to NV GII.4 Sydney.

In this Austrian wedding outbreak, a kitchen worker who had fallen ill from infection with the outbreak strain on October 5 continued to work during preparation of the wedding dinner. His main task was washing dinnerware and flatware, and preparing them for food service implicating frequent hand contact. The outbreak strain possibly entered the kitchen through this symptomatic kitchen worker and spread through hand contact to the environment such as kitchen surfaces and dinner and flatware. The 15 (57 %) of the 26 outbreak cases, which could not be explained by consumption of a wedding specific dish, as shown by the findings of the stratified food-specific analyses, could have been caused by exposure to NV contaminated flatware. Kitchen workers or food handlers are often involved in the spread of NV, which often results in foodborne outbreak (Nicolay et al. 2011; Barrabeig et al. 2010; Schmid et al. 2007, 2011; Ozawa et al. 2007; Teunis et al. 2008), since NV is a highly infectious pathogen with a tiny inoculum (as few as ten virus particles) required for infection (Marshall and Bruggink 2011). The epidemiologically associated mushroom dish could have been contaminated directly by the involved asymptomatic kitchen workers when adding parsley by hand after heating and prior to serving. The mushrooms could also have been contaminated by contact with flatware or dinnerware, previously contaminated by the symptomatic kitchen worker during cleaning. A foodborne NV GII.4 Sydney outbreak in September 2012 in Auckland, New Zealand with 37 cases (attack rate 24.1 %) was due to food, which was manually manipulated after cooking by a food handler who had returned to work prior to 48 h after symptoms had ceased (Thornley et al. 2013). Despite the legal requirement for annual food safety and hygiene training of all food workers in Austria, in our outbreak a kitchen-worker continued working with diarrhoea. Such violations by kitchen workers should give reason for local public health authorities to monitor the adherence to the legal requirement of annual training more vigilantly. Pichler et al. (2014) investigated the level of knowledge on food safety among food handlers from restaurants and various catering businesses in Vienna, Austria from 2011 to 2012 and found that 27 % of food handlers had not participated in any training during the past year. Therefore, our finding, that the ill kitchen worker who continued to work during the wedding dinner had no documented proof of participation in any training during the past year, is not surprising.

We report on the first epidemiologically investigated outbreak due to the NV GII.4 Sydney, a novel NV strain, which has not been observed in Austria before 2012. There was no increase in the severity of disease in any of the cases infected with this new strain, as compared to patients infected with other NV genotypes. The emergence of the novel NV strain did not cause increased seasonal virus activity in Austria. No food safety training was documented for the employees and the kitchen staff’s restroom was lacking operational facilities for hand hygiene. Gender-specific food preferences may pose females at a higher risk of NV gastroenteritis.

## References

[CR1] Arias C, Sala MR, Dominguez A, Torner N, Ruiz L, Martinez A (2010). Epidemiological and clinical features of norovirus gastroenteritis in outbreaks: a population-based study. Clinical Microbiology and Infection.

[CR2] Askovic B, Kirchengast S (2012). Gender differences in nutritional behavior and weight status during early and late adolescence. Anthropologischer Anzeiger.

[CR3] Barrabeig I, Rovira A, Buesa J, Bartolome R, Pinto R, Prellezo H (2010). Foodborne norovirus outbreak: The role of an asymptomatic food handler. BMC Infectious Diseases.

[CR4] Bennett, S., MacLean, A., Miller, R. S., Aitken, C., & Gunson, R. N. (2013). Increased norovirus activity in Scotland in 2012 is associated with the emergence of a new norovirus GII. 4 variant. Euro Surveill, 18(2). 23324428

[CR5] Bernard H, Hohne M, Niendorf S, Altmann D, Stark K (2013). Epidemiology of norovirus gastroenteritis in Germany 2001–2009: eight seasons of routine surveillance. Epidemiology and Infection.

[CR6] Centers for Disease Control & Prevention (2013). Emergence of new norovirus strain GII.4 Sydney–United States, 2012. MMWR. Morbidity and Mortality Weekly Report.

[CR7] Desai R, Hembree CD, Handel A, Matthews JE, Dickey BW, McDonald S (2012). Severe outcomes are associated with genogroup 2 genotype 4 norovirus outbreaks: A systematic literature review. Clinical Infectious Diseases.

[CR8] Division of Viral Diseases, National Center for Immunization and Respiratory Diseases, Centers for Disease Control & Prevention (2011). Updated norovirus outbreak management and disease prevention guidelines. (Practice guideline). MMWR Recommendations and Reports.

[CR9] Fankhauser RL, Monroe SS, Noel JS, Humphrey CD, Bresee JS, Parashar UD (2002). Epidemiologic and molecular trends of “Norwalk-like viruses” associated with outbreaks of gastroenteritis in the United States. Journal of Infectious Diseases.

[CR10] Fekete C, Weyers S, Moebus S, Dragano N, Jöckel KH, Erbel R (2012). Age-specific gender differences in nutrition: Results from a population-based study. Journal of Health Behavior and Public Health.

[CR11] Felsenstein J (1989). PHYLIP—Phylogeny Interference Package (Version 3.2). Cladistics.

[CR12] Fonager, J., Hindbaek, L., & Fischer, T. (2013). Rapid emergence and antigenic diversification of the norovirus 2012 Sydney variant in Denmark, October to December, 2012. Euro Surveilliance, 18(9). 23470017

[CR13] Health Protection Agency (HPA). Update on seasonal norovirus activity. London: HPA, 18 December 2012. http://www.hpa.org.uk/webw/HPAweb&HPAwebStandard/HPAweb_C/1317137436431. Accessed 28 May 2013.

[CR14] Huhulescu S, Kiss R, Brettlecker M, Cerny RJ, Hess C, Wewalka G (2009). Etiology of acute gastroenteritis in three sentinel general practices, Austria 2007. Infection.

[CR15] Lederer, I., & Springer, B. (2010). Noroviren—Jahresbericht 2009 der Nationalen Referenzzentrale. Newsletter. http://www.bmgf.gv.at/home/Schwerpunkte/Krankheiten/Newsletter_Public_Health/Archiv_2011/_Noroviren_Jahresbericht_2010_der_Nationalen_Referenzzentrale_. Accessed 28 May 2013.

[CR16] Lederer, I., & Springer, B. (2011). Noroviren—Jahresbericht 2010 der Nationalen Referenzzentrale. Newsletter. http://www.bmgf.gv.at/cms/home/attachments/8/9/7/CH1305/CMS1299590313499/jb_noroviren_2010.pdf. Accessed 28 May 2013.

[CR17] Lederer, I., & Springer, B. (2012). Noroviren—Jahresbericht 2011 der Nationalen Referenzzentrale. *Newsletter*. http://www.bmg.gv.at/cms/home/attachments/4/0/7/CH1338/CMS1339606686860/noroviren_jb_2011_neu_iii_11062012.pdf. Accessed 28 May 2013.

[CR18] Li J, Predmore A, Divers E, Lou F (2012). New interventions against human norovirus: Progress, opportunities, and challenges. Annual Review of Food Science Technology.

[CR19] Marshall JA, Bruggink LD (2011). The dynamics of norovirus outbreak epidemics: Recent insights. International Journal of Environmental Research and Public Health.

[CR20] Marshall JA, Dimitriadis A, Wright PJ (2005). Molecular and epidemiological features of norovirus-associated gastroenteritis outbreaks in Victoria, Australia in 2001. Journal of Medical Virology.

[CR21] Maunula L, Klemola P, Kauppinen A, Söderberg K, Nguyen T, Pitkänen T (2009). Enteric viruses in a large waterborne outbreak of acute gastroenteritis in Finland. Food and Environmental Virology.

[CR22] Nicolay, N., McDermott, R., Kelly, M., Gorby, M., Prendergast, T., Tuite, G., et al. (2011). Potential role of asymptomatic kitchen food handlers during a food-borne outbreak of norovirus infection, Dublin, Ireland, March 2009. Euro Surveilliance, 16(30). 21813080

[CR23] Oh DY, Gaedicke G, Schreier E (2003). Viral agents of acute gastroenteritis in German children: prevalence and molecular diversity. Journal of Medical Virology.

[CR24] Ozawa K, Oka T, Takeda N, Hansman GS (2007). Norovirus infections in symptomatic and asymptomatic food handlers in Japan. Journal of Clinical Microbiology.

[CR25] Pichler J, Ziegler J, Aldrian U, Allerberger F (2014). Evaluating levels of knowledge on food safety among food handlers from restaurants and various catering businesses in Vienna, Austria 2011/2012. Food Control.

[CR27] Schmid D, Kuo HW, Hell M, Kasper S, Lederer I, Mikula C (2011). Foodborne gastroenteritis outbreak in an Austrian healthcare facility caused by asymptomatic, norovirus-excreting kitchen staff. Journal of Hospital Infection.

[CR28] Schmid D, Stuger HP, Lederer I, Pichler AM, Kainz-Arnfelser G, Schreier E (2007). A foodborne norovirus outbreak due to manually prepared salad, Austria 2006. Infection.

[CR29] Siebenga JJ, Vennema H, Renckens B, de Bruin E, van der Veer B, Siezen RJ (1995). Epochal evolution of GGII.4 norovirus capsid proteins from 1995 to 2006. Journal of Virology.

[CR30] Springer, B. (2009). Noroviren—Jahresbericht 2008 der Nationalen Referenzzentrale. Newsletter, http://www.bmgf.gv.at/home/Schwerpunkte/Krankheiten/Newsletter_Public_Health/Archiv_2010/Noroviren_Jahresbericht_2009_der_Nationalen_Referenzzentrale. Accessed 28 May 2013.

[CR31] Teunis PF, Moe CL, Liu P, Miller SE, Lindesmith L, Baric RS (2008). Norwalk virus: How infectious is it?. Journal of Medical Virology.

[CR32] Thornley CN, Hewitt J, Perumal L, VAN Gessel SM, Wong J, David SA (2013). Multiple outbreaks of a novel norovirus GII.4 linked to an infected post-symptomatic food handler. Epidemiology and Infection.

[CR33] van Beek J, Ambert-Balay K, Botteldoorn N, Eden JS, Fonager J, Hewitt J (2013). Indications for worldwide increased norovirus activity associated with emergence of a new variant of genotype II.4, late 2012. Euro Surveillance.

